# Multifunctional nanoparticle-VEGF modification for tissue-engineered vascular graft to promote sustained anti-thrombosis and rapid endothelialization

**DOI:** 10.3389/fbioe.2023.1109058

**Published:** 2023-01-17

**Authors:** Yalin Liu, Haoyong Yuan, Yuhong Liu, Chunyang Chen, Zhenjie Tang, Can Huang, Zuodong Ning, Ting Lu, Zhongshi Wu

**Affiliations:** ^1^ Department of Cardiovascular Surgery, The Second Xiangya Hospital of Central South University, Changsha, China; ^2^ Engineering Laboratory of Hunan Province for Cardiovascular Biomaterials, Changsha, China; ^3^ Department of Cardiovascular Medicine, The Second Xiangya Hospital of Central South University, Changsha, China; ^4^ Research Institute of Blood Lipid and Atherosclerosis, Central South University, Changsha, China; ^5^ National Health Commission Key Laboratory of Birth Defects Research, Prevention and Treatment, Changsha, China

**Keywords:** small-diameter vascular grafts, REDV peptide, nanoparticles, surface modification, endothelialization

## Abstract

**Purpose:** The absence of a complete endothelial cell layer is a well-recognized reason leading to small-diameter tissue-engineered vascular graft failure. Here we reported a multifunctional system consisting of chitosan (CS), Arg-Glu-Asp-Val (REDV) peptide, heparin, and vascular endothelial growth factor (VEGF) to achieve sustained anti-thrombosis and rapid endothelialization for decellularized and photo-oxidized bovine internal mammary arteries (DP-BIMA).

**Methods:** CS-REDV copolymers were synthesized *via* a transglutaminase (TGase) catalyzed reaction. CS_-REDV_-Hep nanoparticles were formed by electrostatic self-assembly and loaded on the DP-BIMA. The quantification of released heparin and vascular endothelial growth factor was detected. Hemolysis rate, platelets adhesion, endothelial cell (EC) adhesion and proliferation, and MTT assay were performed *in vitro*. The grafts were then tested in a rabbit abdominal aorta interposition model for 3 months. The patency rates were calculated and the ECs regeneration was investigated by immunofluorescence staining of CD31, CD144, and eNOS antibodies.

**Results:** The nanoparticle-VEGF system (particle size: 61.8 ± 18.3 nm, zeta-potential: +13.2 mV, PDI: .108) showed a sustained and controlled release of heparin and VEGF for as long as 1 month and exhibited good biocompatibility, a lower affinity for platelets, and a higher affinity for ECs *in vitro*. The nanoparticle-VEGF immobilized BIMA achieved 100% and 83.3% patency in a rabbit abdominal interposition model during 1 and 3 months, respectively, without any thrombogenicity and showed CD31, CD144, eNOS positive cell adhesion as early as 1 day. After 3 months, CD31, CD144, and eNOS positive cells covered almost the whole luminal surface of the grafts.

**Conclusion:** The results demonstrated that the multifunctional nanoparticle-VEGF system can enhance the anti-thrombosis property and promote rapid endothelialization of small-diameter tissue-engineered vascular grafts. Utilizing nanoparticles to combine different kinds of biomolecules is an appropriate technology to improve the long-term patency of small-diameter tissue-engineered vascular grafts.

## Introduction

Tissue-engineered vascular grafts (TEVGs) have garnered particular attention in research and clinic. The huge clinical demand gave rise to the continuous popularity of TEVGs research. To date, the large diameter TEVGs have yielded significant success in cardiovascular surgery, especially in the treatment of congenital heart diseases. However, the research on small-diameter TEVGs still has a long way to go. Several TEVGs have already been tested in clinical trials for hemodialysis access (Wystrychowski et al., 2014; Lawson et al., 2016) and cavopulmonary conduits (Hibino et al., 2010). While many advances have been achieved, small-diameter TEVGs still face challenges such as incomplete endothelialization, thrombosis, neo-intimal hyperplasia following implantation, and limited function, which can lead to immediate or short-term graft failure after implantation (Lawson et al., 2016; Mi et al., 2018; Bao et al., 2021). To solve these problems, many surface modification technologies have been used to improve anti-thrombosis, endothelial cells(ECs), and endothelial progenitor cells(EPCs) adhesion and proliferation properties of the grafts, but there is still room for improvement (Li et al., 2016; Holzer et al., 2017; Wang et al., 2020).

Biomolecules can be introduced to TEVGs surface by composites (Sevostyanova et al., 2018; Joshi et al., 2020), physical adsorption (Guo et al., 2019), plasma treatment (Gao et al., 2017; Akdogan and Sirin, 2021), and chemical conjugation (Mahara et al., 2015; Zhang et al., 2019). Because of its simple realization, the physical adsorption method for loading biomolecules has received considerable attention. However, the weak electrostatic bonding force will lead to burst release and a limited release cycle (Liu et al., 2019). The situation is the same with plasma treatment, which is also non-covalent bonding in essence. Furthermore, the growth factors are susceptible to the *in vivo* microenvironment without any protection in the blood, which will decrease their bioactivity for vascular antithrombosis and endothelialization (Tan et al., 2011). Chemical conjugation methods are solid enough but always resulted in multi-point fixation of the biomolecules which will affect their bioactivity. Therefore, designing a functional and sustained release structure that can dynamically adapt to the physiological microenvironment that occurs in the blood flow may be an effective method to achieve rapid endothelialization and long-term anticoagulation (Wang et al., 2020).

In our previous study, a controlled release system based on Chitosan and heparin was developed and utilized to modify the lumen surface of decellularized bovine jugular vein grafts. Although high loading capacities and controlled release of vascular endothelial growth factor (VEGF) have been demonstrated, rapid and complete endothelialization was not yet achieved (Devalliere et al., 2018). One of the possible reasons is that we didn’t introduce ECs/EPCs adhesion molecules into the system. The limited ECs sources may limit the speed and level of endothelialization. In addition, the effect of this system on small-diameter vascular grafts has not been studied.

REDV is a fibronectin-derived peptide that has a high binding affinity to α4β1 integrin. Since α4β1 integrin is mainly expressed on ECs and EPCs, REDV possesses the capability of specifically enhancing ECs/EPCs adhesion (Sang et al., 2010; Wei et al., 2011; Mahara et al., 2015). The effects of REDV peptide on vascular graft endothelialization were reported in a few studies. The binding type between the peptide and the lumen surface seems to be crucial for protecting the bioactivity of the peptide. Covalent bonding is helpful for the peptide permanently existing on the graft. However, if the peptide was fixed on the surface by more than one strong chemical bond, the spatial conformation must be changed and must lead to the peptide disfunction. Thus a ‘point-to-point’ covalent bonding should be designed to maximize the peptide’s bio-function.

Transglutaminase (TGase, protein-glutamine γ-glutamyl transferase) is an enzyme that can catalyze a specific acyl transfer reaction between γ-carboxamide groups of peptide-bound glutamine residues and the ε-amino groups of lysine residues within peptides and the primary amino groups of some naturally occurring polyamines (Lu et al., 2010). Chitosan is a natural, biocompatible, and biodegradable cationic polysaccharide the applications of which in tissue engineering and drug delivery have been widely studied. There are plenty of primary amino groups on the side of the chitosan chain, which presages the possibility of covalently binding peptides with chitosan by using TGase. However, there’s little information in the literature about chitosan-peptide copolymer synthesized with TGase as a catalyst.

Decellularized human and animal tissues are extensively used as biomedical materials due to their natural vascular structure and components. The natural architecture of decellularized tissues as well as their diverse structural and functional bioactive molecules makes them potentially advantageous for use in TEVGs. Our previous studies introduced decellularized and photooxidative bovine jugular vein grafts in animal studies and clinical use (Zhu et al., 2017; Devalliere et al., 2018). The grafts were successful in large-diameter vascular grafts. In this study, we choose the bovine internal mammary arteries (BIMA) to match the request of small-diameter vascular grafts and test their potential for research and clinical use.

**FIGURE 1:**
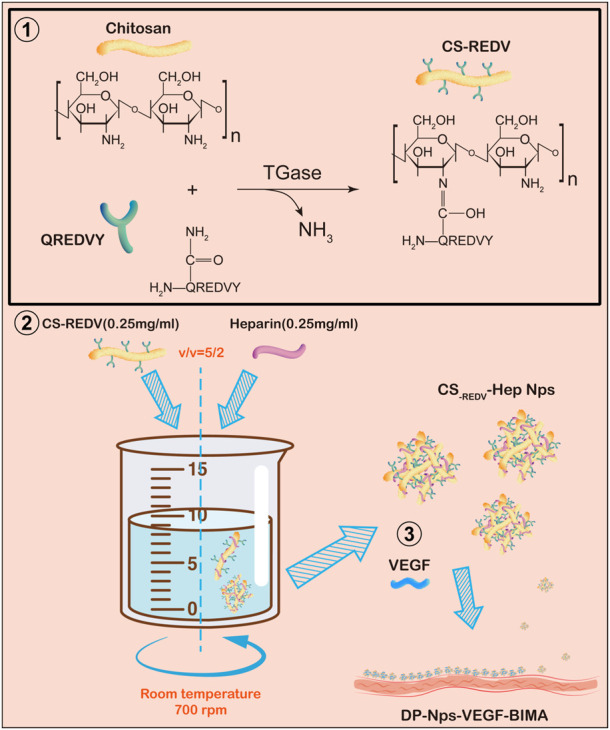
A schematic diagram of this study. Step 1: QREDVY peptides were grafted on chitosan side chain *via* transglutaminase catalysis to synthesize CS-REDV copolymers. Step 2: Mixing the .25 mg/mL CS-REDV solution and .25 mg/mL heparin solution slowly to form CS_-REDV_-Hep nanoparticles. Step 3: Immobilizing the CS_-REDV_-Hep Nps and VEGF onto the Decellularized and photo-oxidized bovine internal mammary artery. And then the grafts were test *in vitro* and *in vivo*.

Here, we introduced a novel multi-functional nanoparticle system containing chitosan, heparin, REDV peptide, and VEGF (Figure 1), and studied the characteristics and bio-functions of the nanoparticle-VEGF immobilized BIMA. The nanoparticle-VEGF system demonstrated good anti-thrombosis, ECs adhesion, and ECs proliferation property, which are crucial for TEVG patency and endothelialization.

## Materials and methods

### Preparation of acellular photo-oxidized vascular grafts

Fresh BIMAs (20 cm in length and 3–4 mm in diameter) were obtained from a local slaughterhouse. After carefully removing the surrounding tissue, the BIMA was perfused in ultrasonic oscillation for 24 h with SDS and Triton-X100 solution (.5% SDS +.5% Triton-X100, Sigma-Aldrich, United States), and 40 U/mL ribozyme (Beijing Dingguo Changsheng Biotechnology Co. Ltd. China) for 12 h. The BIMA was cut into 2 cm segments and treated with 40U/mL ribozyme again, and then soaked into Tris buffer shaking slowly for 6 h at 37°C. The decellularized BIMA was treated with the dye-mediated photo-oxidization procedure as previously described (Zhu et al., 2017). After that, the decellularized and photo-oxidized BIMA (DP-BIMA) were sterilized by 25 KGY γ-ray and stored in 60% alcohol at 4°C.

### CS-REDV copolymer synthesis and preparation of nanoparticles

The amount of 2.5 mg TGase (RHAWN, China) was added to a 100 mL mixed solution of .25 mg/mL chitosan (Zhejiang Golden-Shell Pharmaceutical Co. Ltd. China) and .1 mg/mL QREDVY (Nanjing Peptide Biotech Ltd., China). The enzymatic reaction was performed with magnetic stirring at 300 rpm, 50°C, pH 5.0 for 1 h, and put in boiling water for 10 min to deactivate TGase, and cooled rapidly in ice water. The solution was dialyzed with an 8000 D dialysis membrane (Yuanye Biotechnology Co. Ltd. China) for 3 days and then freeze-dried to get CS-REDV powder. The copolymer was detected by Fourier transform infrared spectroscopy (FTIR, PerkinElmer Co. Ltd., United States) with CS powder as a control. Additionally, the fluorescence intensity of tyrosine was detected by F-4600 FL Spectrophotometer (Serial Number: 2208–007, ROM Version: 5J24000 01, Hitachi, Japan) to quantify QREDVY peptide in the copolymer. Briefly, a standard curve formula of QREDVY fluorescence intensity-concentration was calculated by detecting the fluorescence intensity of gradient concentration QREDVY solution (0, 20, 40, 60, 80, and 100 μg/mL). The fluorescence intensity of .25 mg/mL CS-REDV solution and .25 mg/mL chitosan solution were detected as well. Distilled water and 100 μg/mL QREDVY were used as negative control and positive control, respectively. The excitation wavelength was set as 278 nm, and fluorescence intensity between 280 and 400 nm was scanned. Tyrosine had an emission peak at 310 nm in this condition. Then, the concentration of QREDVY peptide in the .25 mg/mL CS-REDV solution (c) can be calculated. The QREDVY grafting ratio (R), which was defined as the number of QREDVY molecules that grafted on every one hundred repeated units of chitosan, was calculated by the formula below:

$$R=\frac{c/M_{QREDVY}}{\left(0.25-c\right)/M_{CS\;unit}}\times 100\%$$

Among this, R is the grafting ratio of QREDVY. M_QREDVY_ is QREDVY molecular weight, M_QREDVY_ = 898.92. M_CS unit_ is chitosan unit molecular weight, M_CS_ _unit_ = 161.2.

To prepare CS-REDV-Hep nanoparticles, 16 mL .25 mg/mL low molecular weight heparin (5000 D, Suzhou WisMed Pharmaceuticals Co. Ltd. China) was dropwise added into 32 mL .25 mg/mL CS-REDV (pH = 5) with magnetic stirring at 700 rpm, room temperature. The negatively charged heparin molecules and the positively charged CS-REDV molecules formed nanoparticles by electrostatic self-assembly. The size and zeta potential of the CS-REDV-Hep nanoparticles were detected by Series Particle Size and Zeta Potential Analyzer (Malvern Panalytical, Ltd., Spectris, England) and transmission electron microscope (TEM, TecnaiG220S-Twin, FEI, Czech). The unconjugated heparin was detected by the toluidine blue test as previously described (Devalliere et al., 2018).

### Surface modification of DP-BIMA

The DP-BIMAs were treated with sterile EDC/NHS buffer (6.25 mM EDC, 5.00 mM NHS in 40 mL .05 M MES) for 2 h to activate the carboxyl groups. After that, the grafts were transferred into the prepared CS_-REDV_-Hep nanoparticles solution or .25 mg/mL heparin solution and shaken at 37°C water bath for 30 min to get CS_-REDV_-Hep nanoparticles modified grafts (DP-Nps-BIMA) and heparin loaded grafts (DP-Hep-BIMA). Then the luminal surface of the grafts was treated with .25 mL 500 ng/mL VEGF-165 (Cell Signaling Technology, Inc. China) solution before use to get the multi-functional grafts (DP-Nps-VEGF-BIMA or DP-Hep-VEGF-BIMA). Briefly, injecting the VEGF-165 solution into the lumen of the grafts and clamping the two ends to full fill the grafts’ lumen and reacting for at least .5 h. The external surface was also immersed in the VEGF-165 solution. Scanning electron microscope (SEM, Nova NanoSEM230, FEI Electron Optics B.V, Czech) was utilized to detect the surface topology of the grafts.

### Quantification of heparin and VEGF release *in vitro*

1 cm × 1 cm DP-Nps-VEGF-BIMAs were immersed in 2.0 mL PBS buffer solution at 37°C (*n* = 3), and 1.0 mL supernatant was collected and frozen at different time points for detection (Day 0, 1, 4, 7, 14, 21, 28, respectively). 1 mL PBS was replenished for each sample after every collection. The DP-BIMA was modified with heparin and VEGF by EDC/NHS conjugation to get DP-Hep-VEGF-BIMA and used as control. Heparin and VEGF concentrations of the collected supernatant were detected by toluidine blue staining and Human VEGF165 PharmaGenie ELISA Kit(ELISAGenie, Ireland), respectively. The .0075% (wt/wt) toluidine blue solution was made by adding 7.5 mg toluidine blue powder and 200 mg sodium chloride in 100 mL distilled water. Standard heparin solution (0, 20, 40, 60, 80, and 100 μg/mL) was prepared to get a standard curve and standard formula for heparin sample detection. The amount of .3 mL supernatant and .3 mL .0075% toluidine blue solution was added to the 15 mL test tube and mixed well. Then 1 mL of normal hexane was added to extract the heparin-toluidine blue complex (The purple liquid). OD_630nm_ was detected by a microplate reader (Multiskan Sky, Thermo Scientific, United States) for each sample, and was used for calculating heparin concentration by the standard formula. The VEGF concentration of each sample was tested and calculated following the Human VEGF165 PharmaGenie ELISA Kit protocol. The heparin and VEGF release curve was drawn by using the calculated data of different time points. For the heparin release test, the average release rate was calculated by the average daily release of heparin. For the VEGF release test, the percentage of the released VEGF was calculated. The Student’s T-tests were used to find statistical differences between the two groups at different time points.

### Biocompatibility and bioactivity of the DP-Nps-VEGF-BIMA

#### Platelets adhesion test

Fresh anticoagulant blood from volunteers was centrifuged at 650 rpm for 10 min, and the upper plasma was reserved for the next centrifugation at 3,000 rpm for 10 min to get platelet-rich plasma. DP-Nps-VEGF-BIMA, DP-BIMA, and fresh native aorta pieces were immersed in 20 mL platelet-rich plasma separately and oscillated at 37°C, 60 rpm in a constant temperature air bath for 2 h and washed with PBS for 3 times (*n* = 6). After that, the adhered platelets were counted randomly by SEM (×5000 magnified view, 10 views for each piece).

#### ECs adhesion and proliferation test

The cell-culture slides were coated with poly-lysine in order to modify them similarly to vascular grafts. Human umbilical vein endothelial cells (HUVECs, EA. HY926 cells) were cultured in DMEM cell culture medium (G4510, Servicebio, China) with 10% fetal bovine serum (FBS, G8001, Servicebio, China), 37°C, 5% CO_2_ concentration in a cell incubator. 2×10^4^ cells were added onto every slide and cultured for 5 days (*n* = 6). On the 1st, 3rd, and 5th day, we stained the living cells with 5-(and-6)-Carboxy-2′,7′-dichlorofluorescein Diacetate [5(6)-CDCFDA, US Everbright Inc., United States] and exposed them through a fluorescence inverted microscope (Eclipse Ti2-U, Nikon, Japan). Analysis of variance for statistics was taken to compare the results between different groups.

#### Hemolysis test

Two mini liters of fresh anticoagulant blood from volunteers were diluted with 2 mL of normal saline for use. DP-Nps-VEGF-BIMA pieces were immersed in 10 mL of normal saline at 37°C, 60 rpm for 30 min. The positive and negative control group was 10 mL distilled water, and 10 mL normal saline without DP-Nps-VEGF-BIMA pieces, respectively. A .2 mL diluted blood sample was added in each group and was oscillated at 37°C, 60 rpm in a constant temperature air bath for 1 h and then centrifuged at 4°C, 3,000 rpm for 10 min. Then we detected optical density (OD) of the supernatant of each sample at 545 nm with a microplate spectrophotometer (Multiskan SkyHigh, ThermoFisher Scientific, United States) and calculated the hemolysis rate. The results were considered valid when there’s no hemolysis was observed in the negative control, and a hemolysis rate of less than 5% was considered qualified according to the National Pharmaceutical Industry Standard (YY/T 1651.1-2019).

#### MTT assay

DP-Nps-VEGF-BIMA pieces were immersed in 10 mL of normal saline at 37°C, 80 rpm for 24 h (*n* = 3). The supernatant was diluted at 1:1, 1:2, 1:3, 1:4, and 1:5 with normal saline to get gradient diluent (*n* = 3). 2000 HUVECs, 2.7 mL diluent, and .3 mL fetal bovine serum per hole were added into a 96-well plate and cultured for 5 days. The number of living cells was detected by MTT colorimetric method.

### *In vivo* experiments

All animal experiments were conducted in accordance with the Guidelines for Animal Experiments established by Central South University. The protocol was approved by the Committee on the Ethics of Animal Experiments of The Second Xiangya Hospital, Central South University.

#### Animal model

All the experiments were approved by the Experimental Animal Welfare Ethics Committee of the Second Xiangya Hospital of Central South University and followed the Guide for Care and Use of Laboratory Animals. The DP-Nps-VEGF-BIMA and DP-BIMA (3 mm in diameter and 2 cm in length) were implanted in the infrarenal abdominal aortas of New Zealand white rabbits (male; weight: 3.0–3.5 kg; age: 6–8 months). Briefly, the animals were anesthetized with 1% pentobarbital sodium (30 mg/kg, injected *via* ear vein). The amount of 1 mL .1 g/mL antibiotics (Cefoperazone Sodium Sulbactam Sodium) and 20 mL glucose saline (10% glucose: .9% normal saline was 2:1) were injected before surgery. The abdominal aorta was exposed through the left lateral abdomen incision. Whole-body heparinization by injection of 1.5 mL 1250 U/mL sodium heparin was needed to avoid thrombosis. The prepared vascular grafts were measured and implanted by end-to-end anastomosis after the native aorta was excised. After surgery, 1 mL .1 g/mL antibiotics and an appropriate amount of glucose saline were injected. Antibiotics were also used one time per day for at least 3 days. Abdominal aorta ultrasound exams were performed at different time points to check the patency of the implanted grafts (Day 1, 4, 7, 14, 1 month, and 3 months). The patency rate of 7 days (*n* = 12), 1 month (*n* = 6), and 3 months (*n* = 6) was calculated respectively. The animals were euthanized by overdose anesthesia (1% pentobarbital sodium, 100 mg/kg, injected *via* ear vein) on day 7 and 3 months (*n* = 6), and the implanted grafts were collected and stored for the following detection.

#### Histochemical and immunofluorescence staining

The explanted grafts were split into three parts, the proximal segment, the middle segment, and the distal segment. The middle segments were fixed by 4% v/v paraformaldehyde, dehydrated by 15% and 30% w/v sucrose solution, and embedded with OCT. Frozen sections 8 μm thick were used for histochemical and immunofluorescence staining. Histochemical staining included H&E staining (Servicebio Ltd., China). Immunofluorescence staining were performed by CD31(GeneTex, GTX34489), CD144(Abcam, ab282277), and eNOS(Abcam, ab76198) antibodies to evaluate endothelialization. DAPI (Servicebio, G1012) was used to counterstain the cell nucleus. The mean fluorescence intensity was calculated by ImageJ to quantify CD31, CD144, and eNOS expression.

### Statistical analysis

The mean ± standard deviation (SD) was used to characterize the data. The student’s t-test was used for heparin and VEGF release, and single-factor analysis of variance was used to express the statistical analyses for the platelets adhesion test, ECs adhesion test, MTT assay, and fluorescent intensity. *p* < .05 indicated statistical significance.

## Results

### Characteristics of DP-Nps-VEGF immobilized BIMA

**FIGURE 2:**
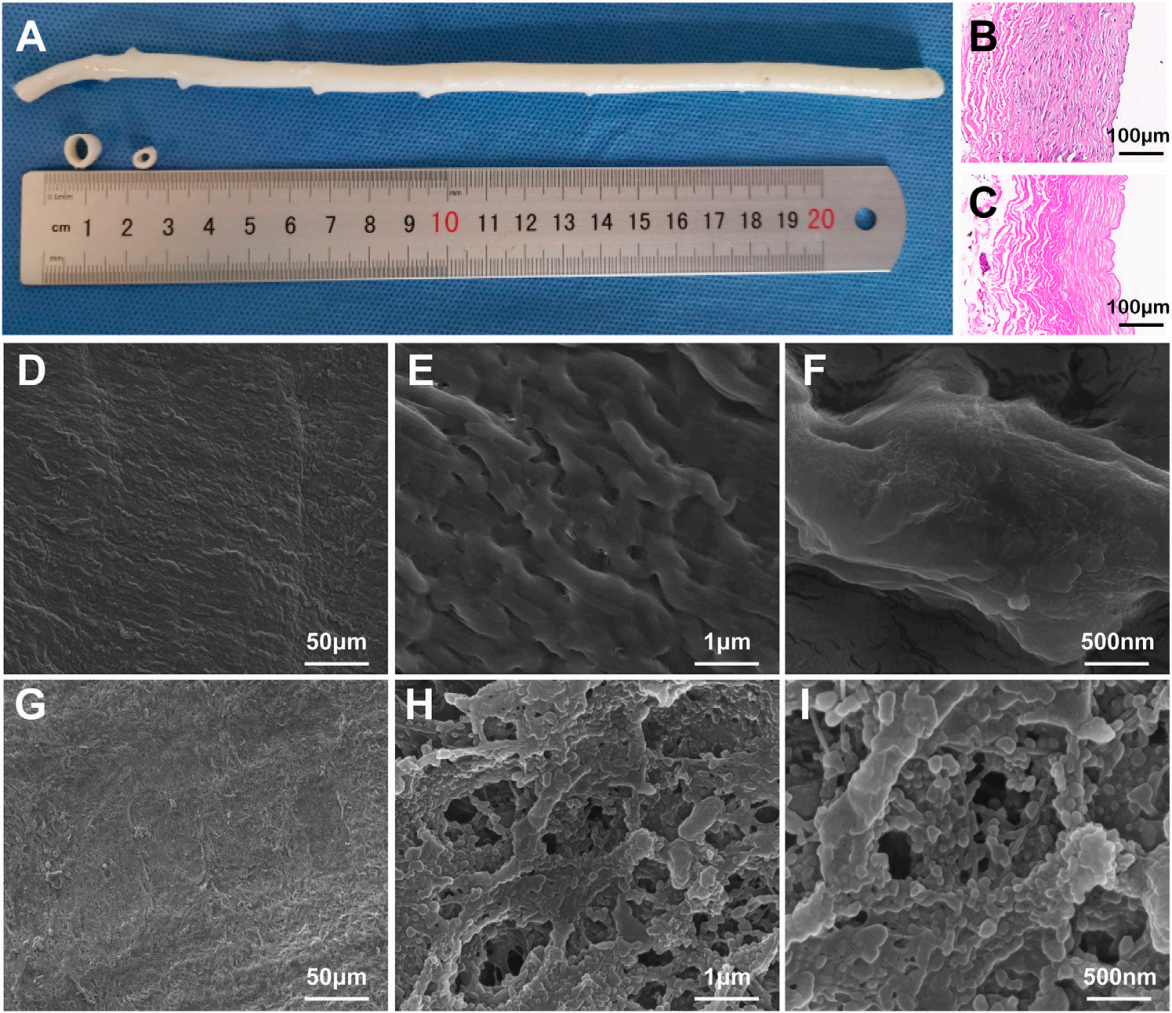
The characteristics of bovine internal mammary arteries (BIMA). **(A)** is the general view of fresh BIMA. **(B,C)** are the H&E staining of fresh BIMA and decellularized and photo-oxidized BIMA (DP-BIMA). **(D–F)** are the SEM images of fresh BIMA. **(G–I)** are the SEM images of CS-REDV-Nps-VEGF modified DP-BIMA. Scale bars are indicated in each image.

The fresh BIMA was shown in Figure 2A. Here we exhibited a longitudinal view of BIMA which was longer than 20 cm, and the cross-section of the proximal and distal BIMA segments which were 2.5 and 4.5 mm in diameter, respectively. The H&E staining images of BIMA before and after decellularization were shown in Figure 2B and Figure 2C, respectively. The SEM images of native BIMA were shown in Figures 2D–F. The luminal surface of native BIMA was not smooth, but with many grooves and ridges. Figures 2G–I showed the luminal surface of DP-Nps-VEGF immobilized BIMA, and we found it more porous than untreated BIMA. The nanoparticles arranged along the fibers and covered almost the whole luminal surface (Figures 2G–I).

### REDV grafting ratio and characterization of nanoparticles

**FIGURE 3:**
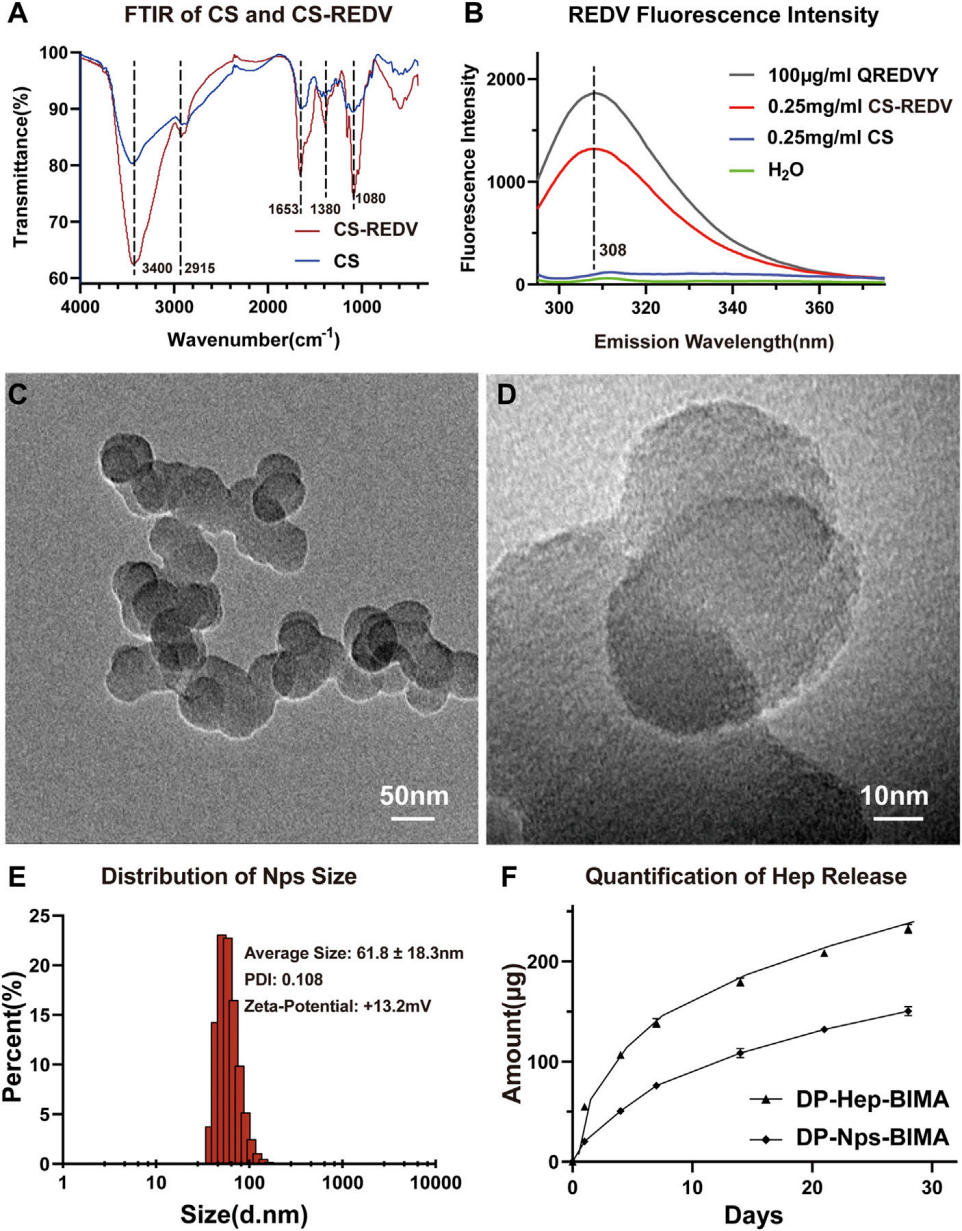
The characteristics of CS_-REDV_-Hep nanoparticles. **(A)** is the Fourier transform infrared spectroscopy curve of chitosan and CS-REDV copolymers. **(B)** is the fluorospectro photometer results of different solutions. H_2_O had no emission wave and was used as a negative control. .25 mg/mL CS solution had no emission wave as well. **(C,D)** are TEM images of the CS_-REDV_-Hep nanoparticles. **(E)** is the characteristics of the nanoparticles (*n* = 3). **(F)** showed the quantification of heparin release in PBS (*n* = 3).

The FTIR and fluorospectro photometer results were shown in Figure 3. According to the FTIR characteristics of chitosan and CS_-REDV_ powder, the FTIR absorption of CS_-REDV_ was enhanced at 1,653 cm^−1^, which represented the Schiff base (C=N) stretching. The absorption at 3,400 cm^−1^ was strengthened, which indicated the increase of hydroxyl groups and amino groups. The CS_-REDV_ group presented enhancement absorption peak at 1,380 and 1,080 cm^−1^, corresponding to C-N and C-O, respectively (Figure 3A). The fluorescence intensity of CS_-REDV_ solution, chitosan solution, distilled water, and QREDVY solution was shown in Figure 3B. Chitosan didn’t show any emission peak between 280 nm and 400 nm wavelength. Instead, the CS_-REDV_ solution showed an emission peak at 308 nm wavelength and the fluorescence intensity was about 70% of the positive control group. The results determined that the synthesis of CS_-REDV_ copolymers was successful. Additionally, the REDV grafting ratio was 6.83% according to the fluorospectro photometer results, which indicated that there were about 7 QREDVY peptides for every 100 repeat units of chitosan (Figure 3B).

The particle size, polymer dispersity index (PDI), and zeta-potential of CS_-REDV_-Hep Nps were shown in Figure 3. Figures 3C, D were the TEM images of the nanoparticles, and Figure 3E was the Series Particle Size and Zeta Potential Analyzer report of the nanoparticles, which showed an average particle size of 61.8 ± 18.3 nm, a PDI of .108, and a Zeta-potential of +13.2 mV. We tested the nanoparticle samples that were stored at 4°C for 1 month. The nanoparticle suspension was stable at 4°C for 1 month without aggregation, with no significant differences of the three parameters above. We detected free heparin in the nanoparticle suspension by toluidine blue staining, the results indicated that there was no free heparin in the CS_-REDV_-Hep Nps suspension, which meant that all the heparin we added in was loaded in the nanoparticles. The accumulated release of heparin was shown in Figure 3F and Table 1, the speed of heparin release was much faster in the DP-Hep-BIMA group than in the nanoparticle group. CS_-REDV_-Hep Nps showed consistent release of heparin in the first month.

**TABLE 1:** *In vitro* release assay of Heparin.

Days	DP-Hep-BIMA (*n* = 3)		DP-Nps-BIMA (*n* = 3)		*p* value
	Amount (μg)	Release rate (μg/d)	Amount (μg)	Release rate (μg/d)	
1	54.99±1.99	54.99±1.99	20.24±0.59	20.24±0.59	<0.001
4	106.76±1.94	17.26±1.28	50.67±0.86	10.14±0.31	<0.001
7	138.55±4.30	10.60±1.06	75.92±3.01	8.42±0.73	0.042
14	172.88±3.22	4.90±0.65	108.53±4.46	4.66±0.67	0.672
21	181.69±5.22	1.26±1.19	132.00±2.00	3.35±0.60	0.053
28	186.98±6.71	0.76±0.21	150.40±4.43	2.63±0.36	0.001

**Notes:** The amount and speed of heparin release were shown in table 1. Release rate indicated the daily released heparin during each time interval. *P*<0.05 indicated statistical difference.

### Quantification of VEGF loading and release *in vitro*

**FIGURE 4:**
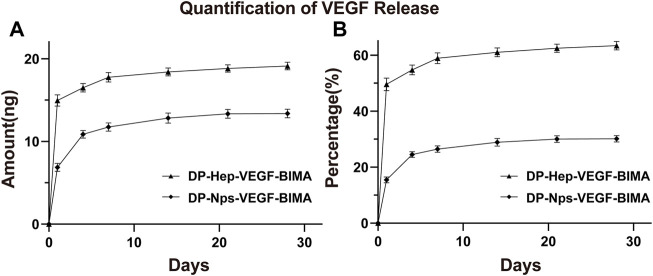
Quantification of VEGF release in amounts **(A)** and in release percentage **(B)**, *n* = 3.

The amount of VEGF loaded on the nanoparticles group was 44.39 ± .30 ng/cm^2^, which was almost 1.5 times of the Hep-VEGF group (30.14 ± .07 ng/cm^2^), *p* < .001. Figures 4A, B, and Table 2 showed the release of VEGF in amount and in proportion, respectively. The release of VEGF from the grafts *via* nanoparticle or heparin immobilization was analyzed over 28 days. VEGF exhibited a burst release during the first 4 days, while the Hep-VEGF group showed a much high amount and burst release speed of VEGF, compared with the CS_-REDV_-Hep Nps-VEGF group. CS_-REDV_-Hep Nps-VEGF group released ∼30% at 28 days, whereas the Hep-VEGF group released more than 60% at the same time (Figure 4B; Table 2).

**TABLE 2:** *In vitro* release assay of VEGF.

Days	DP-Hep-VEGF-BIMA (*n* = 3)		DP-Nps-VEGF-BIMA (*n* = 3)		*p* value
	Amount (ng)	Percentage (%)	Amount (ng)	Percentage (%)	
1	14.95±0.67	49.61%	6.87±0.47	15.47%	<0.001
4	16.50±0.52	54.74%	10.87±0.47	24.49%	<0.001
7	17.76±0.57	58.91%	12.64±0.50	28.48%	<0.001
14	18.39±0.48	61.02%	13.72±0.60	31.91%	<0.001
21	18.83±0.45	62.46%	14.58±0.48	32.84%	<0.001
28	19.12±0.46	63.43%	15.41±0.53	33.82%	<0.001

**Notes:** The amount and percentage of VEGF release were shown in table 2. *P*<0.05 indicated statistical difference.

### Biocompatibility of the CS_-REDV_-Hep-Nps-VEGF immobilized TEVG

The results of the hemolysis test were shown in Supplementary Table S1. Dnc <.03 and Dpc = .80 ± .03 manifested the test was effective, and the rate of the nanoparticle group was .05%, which indicated good hemocompatibility of the DP-Nps-VEGF-BIMA.

**FIGURE 5:**
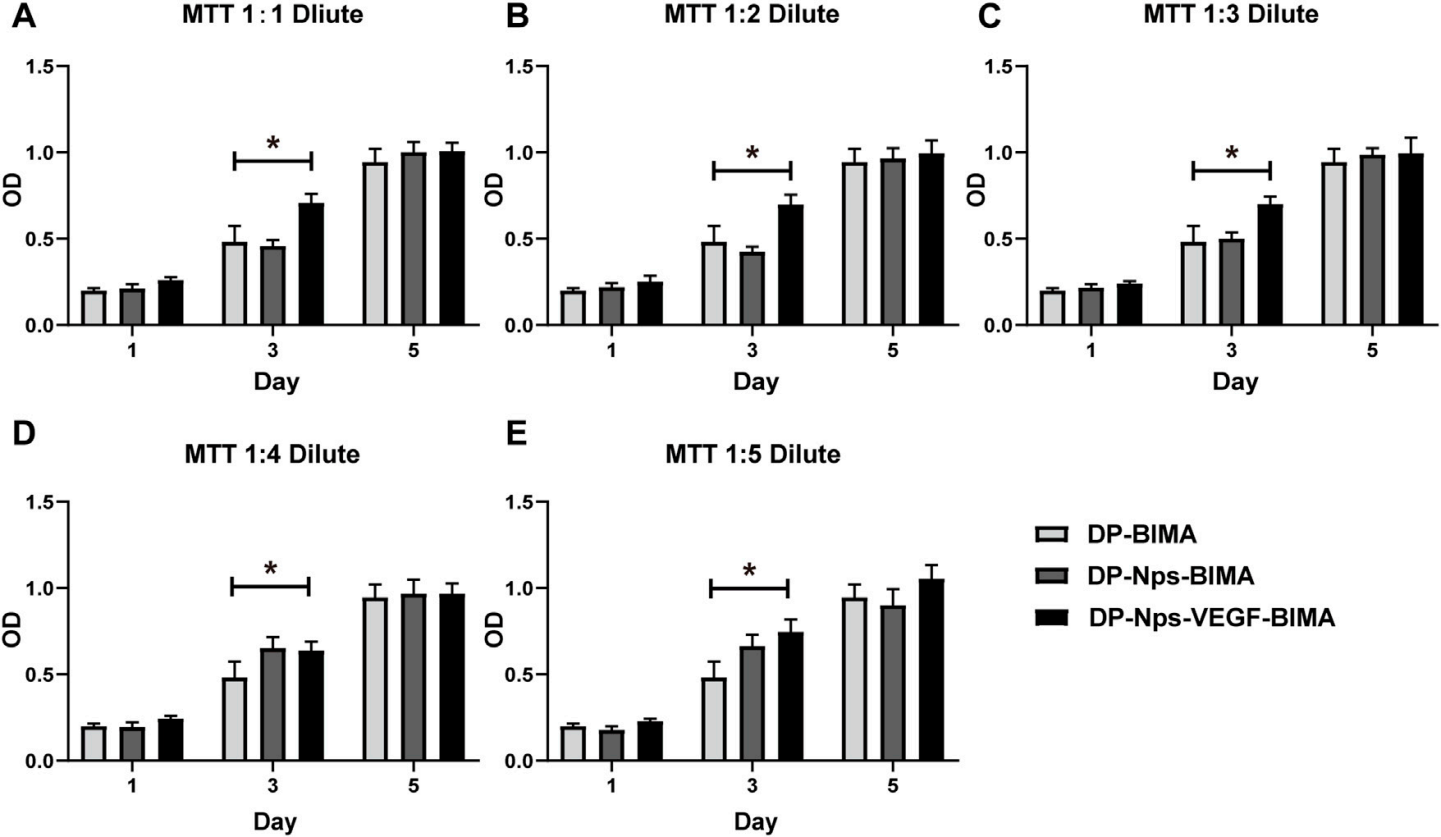
The results of MTT assay with 1:1 diluent **(A)**, 1:2 diluent **(B)**, 1:3 diluent **(C)**, 1:4 diluent **(D)**, and 1:5 diluent **(E)**, *n* = 3. * indicated statistical difference (*p* < .05).

The results of the MTT assay showed that the CS_-REDV_-Hep nanoparticles have no cytotoxicity since there was no significant difference between the nanoparticle group and the control group (Figures 5A–E). Interestingly, The DP-Nps-VEGF-BIMA group had a little higher cell proliferation on day 1, and much higher cell proliferation on day 3, whereas there was no statistical significance on day 5 (Figures 5A–E). That may due to the bioactivity of VEGF in promoting ECs proliferation. The effect of VEGF was more remarkable on day 3. On day 5, VEGF-induced cell proliferation might be limited by the consumption of DMEM.

### Bioactivity of the CS_-REDV_-Hep-Nps-VEGF system

#### Platelet adhesion test

**FIGURE 6:**
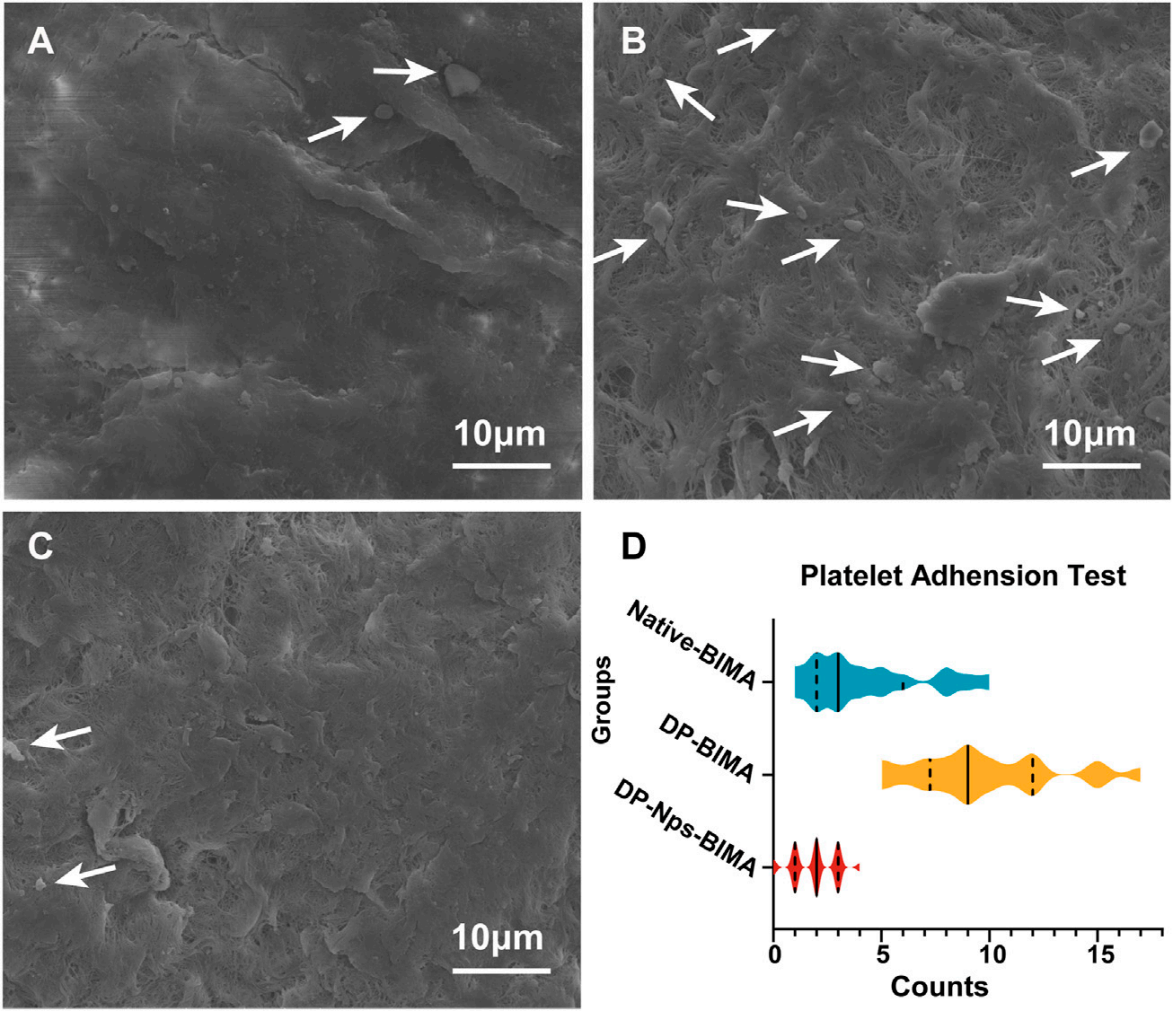
The results of platelets adhesion assay for different groups. **(A)** is the native BIMA, **(B)** is the DP-BIMA, and **(C)** is the DP-Nps-VEGF BIMA. The white arrows indicate the adhered platelets on the luminal surfaces of each groups. **(D)** is the statistical graph which indicates the quartiles of the three groups, the solid line indicates the median and the dotted lines indicate the first and third quartile (*n* = 6).

The amount of adhered platelets on the BIMA surface was 4.26 ± 2.75, 9.80 ± 3.30, 1.92 ± 1.04, for Native-BIMA, DP-BIMA, and DP-Nps-BIMA, respectively (Figure 6). The fresh BIMA had a natural anti-platelets ECs layer and exhibited a low affinity of platelets (Figure 6A). The DP-Nps-BIMA group showed similar anti-platelets adhesion property with native BIMA (Figures 6A, C). That was due to the bioactivity of heparin. However, the DP-BIMA had neither ECs layer nor heparin immobilization and showed a high affinity of platelets (Figure 6B). The violin diagram shows the amounts and distribution of the adhered platelets on Native-BIMA, DP-BIMA, and DP-Nps-BIMA (Figure 6D). The results reflected the probable density of the number of adhered platelets per unit area of BIMA pieces to a certain extent. The quartiles of the Native-BIMA were (2, 3, 6), and increased to (7, 9, 12) for DP-BIMA due to collagen exposure, while it was (1, 2, 3) DP-Nps-BIMA. This indicated that heparin successfully prevented most platelets adhesion and would make DP-NPs-BIMA more anti-thrombotic *in vivo*.

#### ECs adhesion and proliferation assay

**FIGURE 7:**
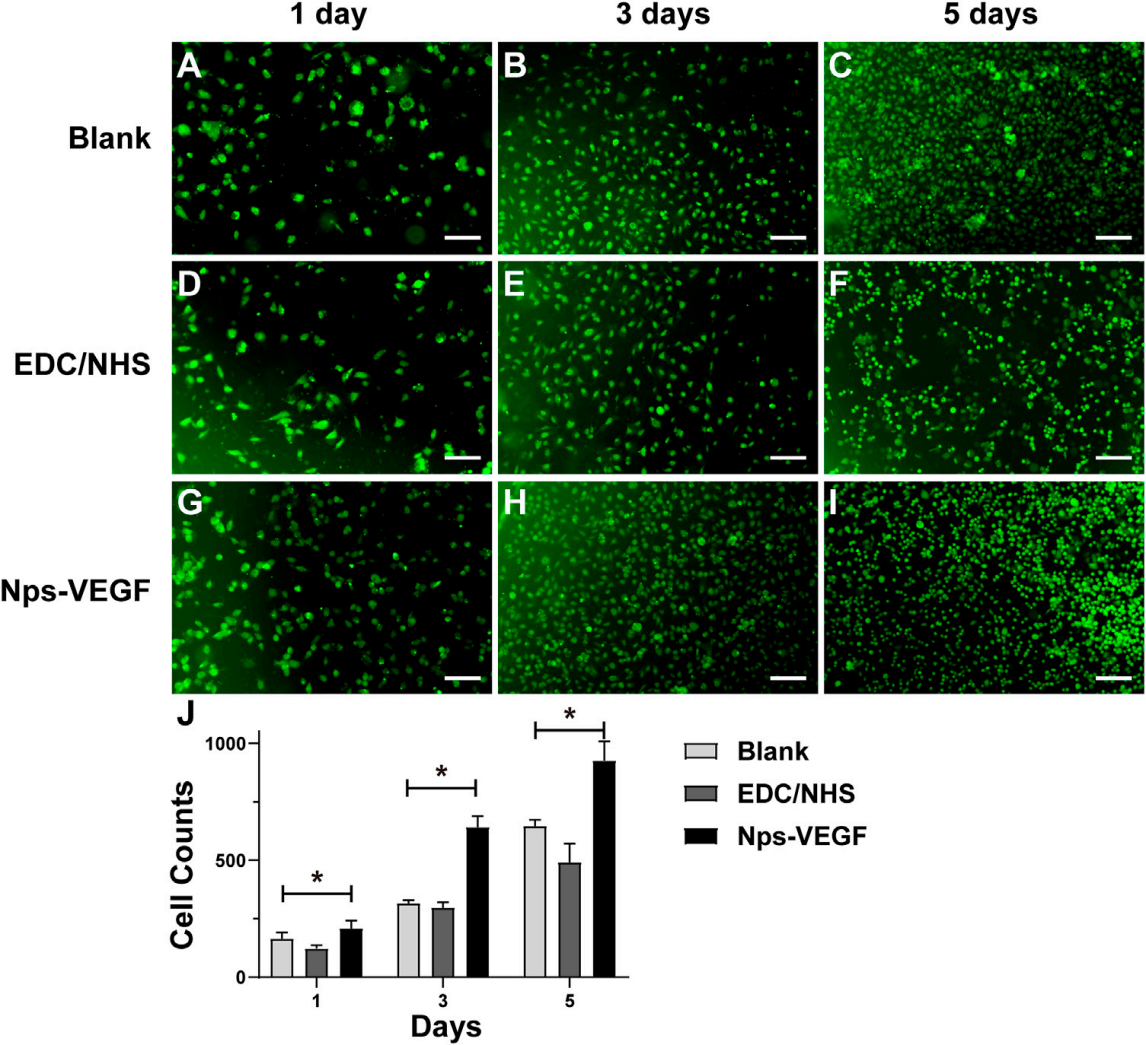
The results of HUVECs adhesion and proliferation assay. **(A–I)** are optical microscope pictures of the three groups on different time points. **(J)** is the statistical graph of cell counts on day 1, day 3, and day 5 (*n* = 6). * indicated statistical difference(*p* < .05).

The HUVECs were seeded onto cell culture slides immobilized with different molecules to test the affinity and proliferation properties of ECs. As shown in Figures 7A–C, the cell counts of the control group on day 1, day 3, and day 5 indicated the ECs proliferation in DMEM cell culture medium with 10% FBS. The results of EDC/NHS control group were shown in Figures 7D–F. Figures 7G–I exhibited the cell counts of the nanoparticle group on day 1, day 3, and day 5, respectively. Figure 7J was the statistical image of cell counts for each group. As shown in Figures 7A, D, G, the ECs counts on day 1 indicated the adhesion property for each group, and the nanoparticle group showed a higher affinity for adhering ECs. The cell counts of the nanoparticle group increased faster on day 3 and day 5 as well (Figures 7B, E, H for day 3; Figures 7C, F, I for day 5; Figure 7J for statistical image), and might be due to the effect of VEGF released from the nanoparticles. The results demonstrated that the CS-REDV-Hep-Nps-VEGF system had a high affinity and strong promoting effect for ECs adhesion and proliferation.

### Rabbits abdominal aorta interposition model and *in vivo* regeneration of ECs

**FIGURE 8:**
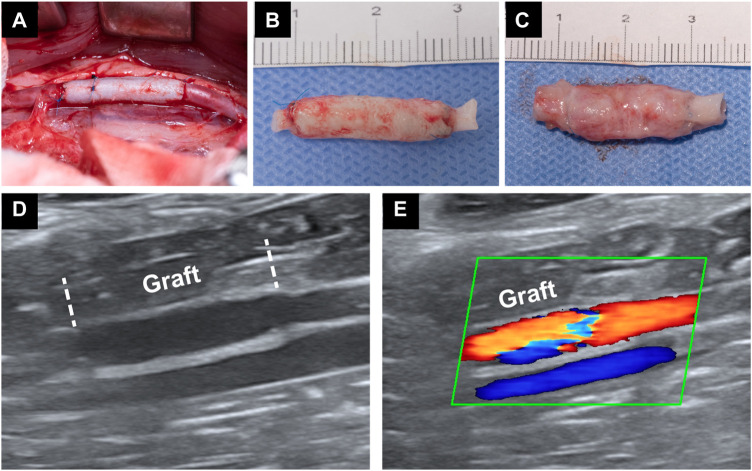
Rabbit abdominal aorta interposition model. **(A)** is a photo of the implanted graft immediately after the surgery. **(B)** is a photo of a graft which was implanted for 1 week. **(C)** is a photo of a graft which was implanted for 3 months. **(D,E)** are the Doppler ultrasound images of an implanted graft.

**FIGURE 9:**
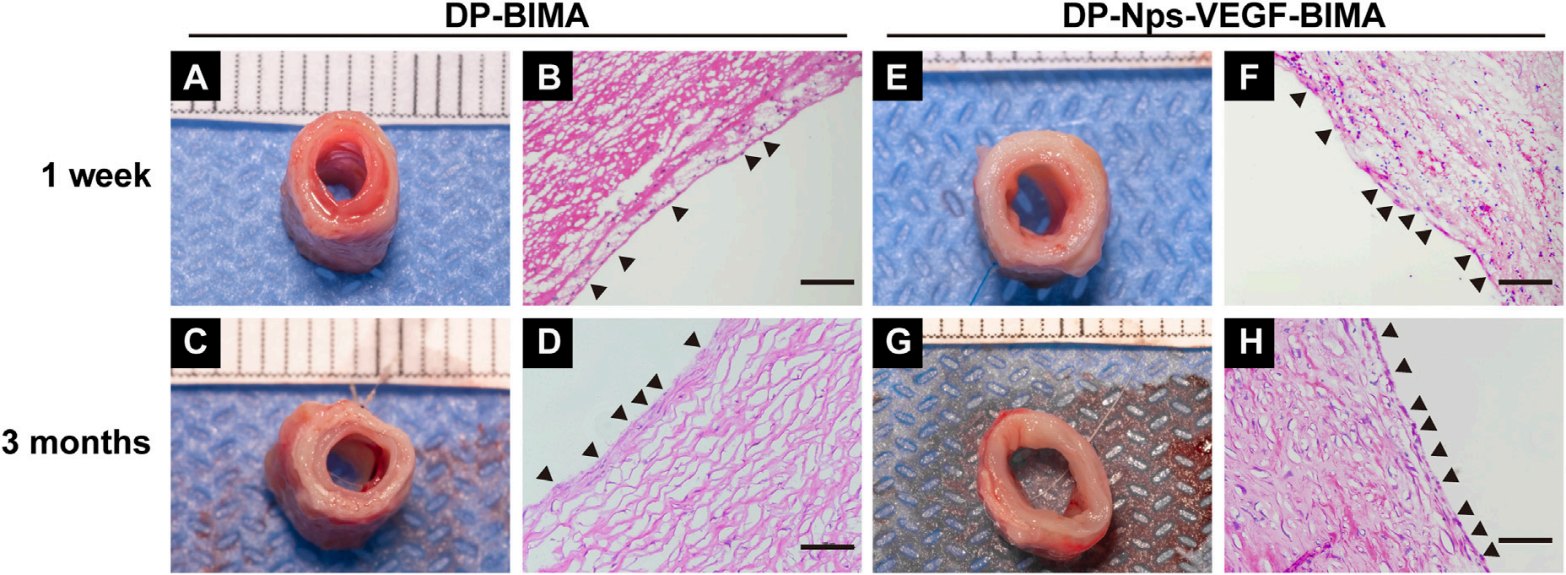
The photos and H&E images of the middle segments of the implanted DP-BIMA **(A–D)** and DP-Nps-VEGF-BIMA **(E–H)**. The black triangles indicates the ECs-like cells.

The abdominal aorta interposition model was successfully performed in the DP-BIMA group and the DP-Nps-VEGF-BIMA group (Figure 8A). After 1 week and 3 months, the grafts were collected and tested (Figures 8B, C). Ultrasound exams were performed at specific time points (Figures 8D, E). The patency rates of different time points were shown in Table 3. The results showed that 2/12 graft was occluded in the DP-BIMA group in 1 week. In the long-term group of DP-BIMA, there were 2/6 and 3/6 grafts were occluded in 1 and 3 months, respectively. In comparison, there were 1/12 grafts of the DP-Nps-BIMA group occluded in 1 week, whereas the six long-term samples kept patent in 1 month and showed a higher patency rate of 5/6 in 3 months compared with DP-BIMA. However, we found 1 nanoparticle immobilized graft among the long-term samples was almost occluded at day 7 (Supplementary Figures S1A–C). The neointimal of this graft was thicker, both at the middle segment and the anastomosis (Supplementary Figure S1). We also found that even the patent grafts had a neointimal formation of varying thickness, but became dense and stable after 3 months of reconstruction *in vivo* (Figure 9). The H&E staining also exhibited a similar saturation (Figure 9). In addition, we didn’t observe any dilation or aneurysm happening among the two groups.

**TABLE 3:** The patency rates of different time points.

Groups	Patency rates (%)			
	1 day	1 week	1 month	3 months
DP-BIMA	100% (12/12)	83.3% (10/12)	66.7% (4/6)	50% (3/6)
DP-Nps-BIMA	100% (12/12)	91.7% (11/12)	100% (6/6)	83.3% (5/6)

**Notes:** DP-BIMA=Decellularized and photo-oxidized bovine internal mammary arteries. DP-Nps-VEGF-BIMA=Nanoparticle and VEGF immobilized DP-BIMA. The 1 day and 1 week samples included the short-term and long-term samples that were sacrificed at 1week and 3 months after implantation. The 1 month and 3 months samples included only long-term samples that were fed for 3 months after implantation.

H&E staining showed good cell infiltration after 3 months of implantation, and we found a complete layer of ECs-like cells on the luminal surface of the nanoparticle and VEGF immobilized grafts (Figure 9H). The DP-BIMA group showed less cell infiltration and an incomplete luminal cell layer (Figure 9D). At 1 week, the DP-Nps-BIMA group showed better cell infiltration as well (Figure 9F). Also, a number of cells covered part of the luminal surface of the grafts (Figure 9F), whereas there were few cells infiltrated in DP-BIMA grafts (Figure 9B).

### Endothelialization of the grafts

**FIGURE 10:**
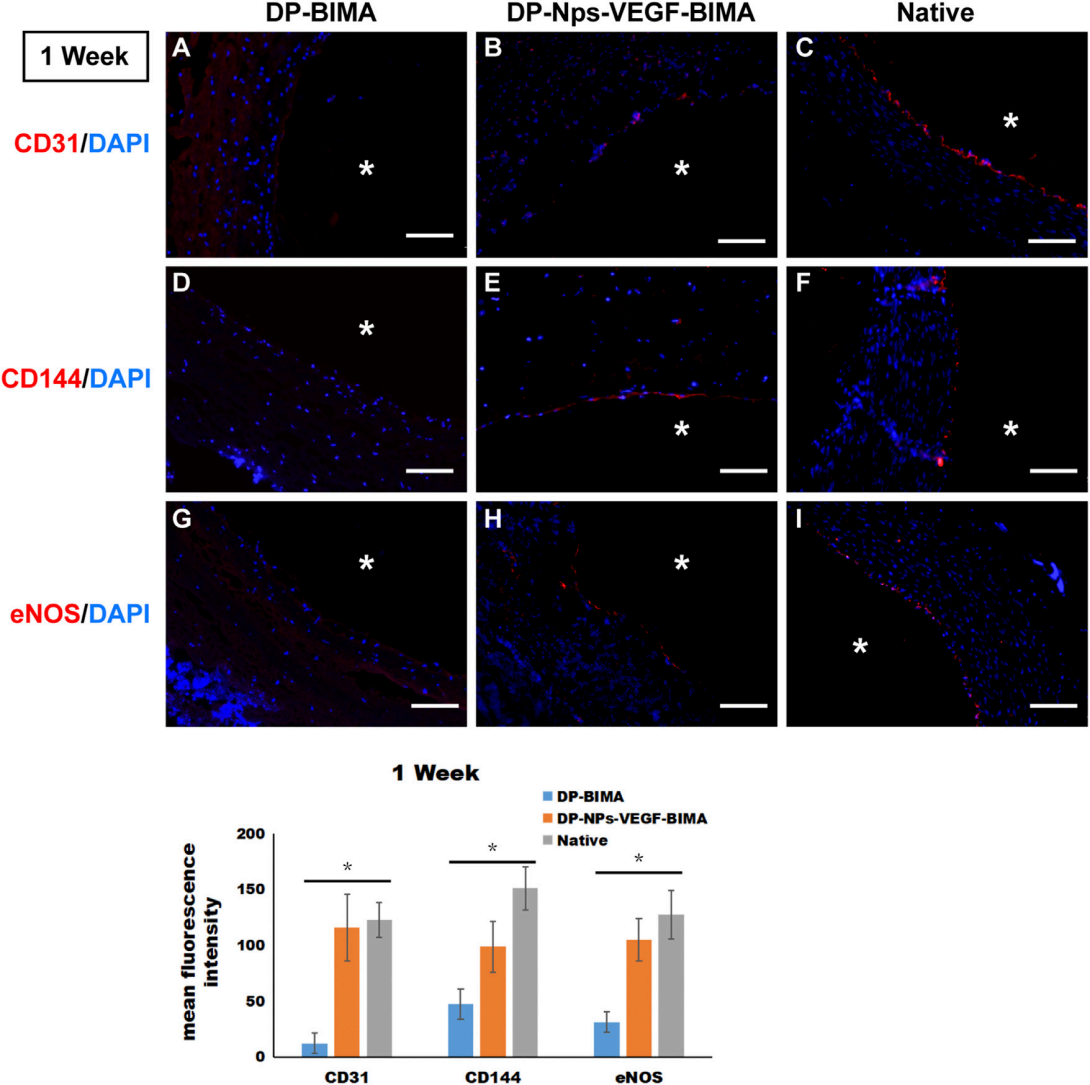
The immunofluorescent staining results of CD31, CD144, and eNOS. Samples are native rabbit aorta, and the grafts of the two groups which were implanted for 1 week. **(A–C)** were the staining of CD31 and DAPI for DP-BIMA, DP-Nps-VEGF-BIMA, and native rabbit aorta, respectively. **(D–F)** were the staining of CD144 and DAPI for DP-BIMA, DP-Nps-VEGF-BIMA, and native rabbit aorta, respectively. **(G–I)** were the staining of eNOS and DAPI for DP-BIMA, DP-Nps-VEGF-BIMA, and native rabbit aorta, respectively. * indicates the lumen of the grafts. Scale bars indicate 100 μm.

**FIGURE 11:**
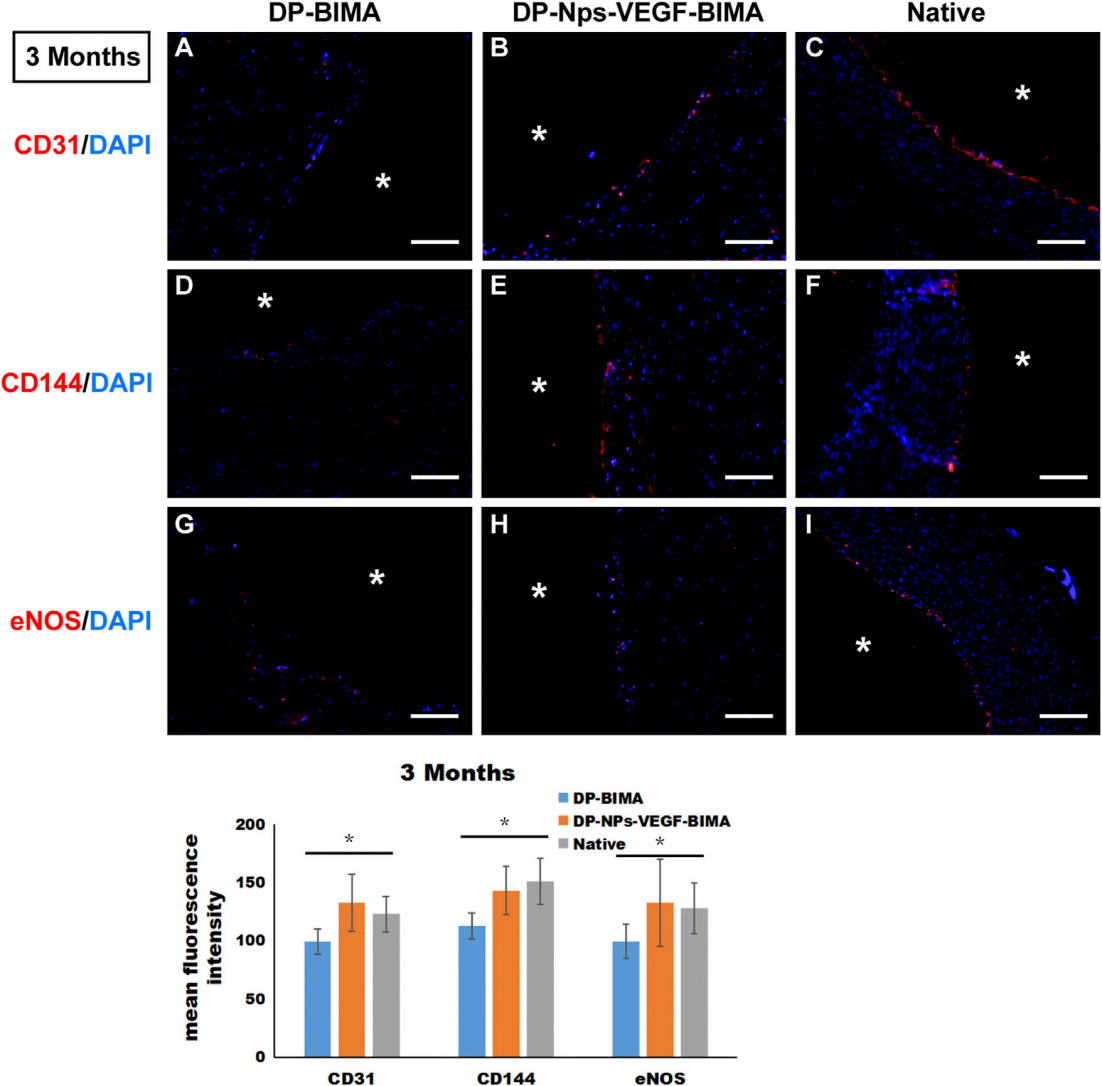
The immunofluorescent staining results of CD31, CD144, and eNOS. Samples are native rabbit aorta, and the grafts of the two groups which were implanted for 3 months. **(A–C)** were the staining of CD31 and DAPI for DP-BIMA, DP-Nps-VEGF-BIMA, and native rabbit aorta, respectively. **(D–F)** were the staining of CD144 and DAPI for DP-BIMA, DP-Nps-VEGF-BIMA, and native rabbit aorta, respectively. **(G–I)** were the staining of eNOS and DAPI for DP-BIMA, DP-Nps-VEGF-BIMA, and native rabbit aorta, respectively.* indicates the lumen of the grafts. Scale bars indicate 100 μm.

CD31, CD144, and eNOS antibodies were utilized to detect ECs at 1 week and 3 months, and the results were shown in Figures 10, 11, respectively. The native rabbit abdominal aorta as a positive control was shown in Figure 10C for these three antibodies, respectively. At 1 week, the DP-BIMA group showed no CD31, CD144, or eNOS positive cells, which indicated that there was no ECs/EPCs adhesion in this group. In comparison, the DP-Nps-VEGF immobilized grafts showed some CD31, CD144, and eNOS positive cells at 1 week, although the luminal surface was partially covered with these ECs-like cells (Figure 10). After 3 months, we found a small group of sporadic ECs-like cells on the luminal surface of DP-BIMA with positive staining of CD31, CD144, and eNOS. Whereas in the DP-Nps-VEGF-BIMA group, the ECs-like cells covered almost all the luminal surface of the grafts, which demonstrated a complete endothelialization during 3 months (Figure 11).

Interestingly, we detected ECs-like cells on the DP-Nps-VEGF immobilized grafts as early as the first day after implantation (Supplementary Figure S2). However, in the DP-BIMA group, there were no positive stained cells found at that early time point. These findings demonstrated that the CS-REDV-Hep-VEGF system can capture ECs/EPCs from circulation immediately after the grafts were implanted, and promoted complete endothelialization *in vivo*.

## Discussion

In this study, we successfully synthesized chitosan-REDV copolymer and CS-REDV-Hep nanoparticles which can efficiently load VEGF. In addition, a controlled release of heparin and VEGF was achieved. The CS-REDV-Hep NPs and VEGF composed a multi-functional system that contained anti-thrombosis, ECs/EPCs adhesion, and ECs/EPCs proliferation properties. These functions are of great significance for maintaining TEVG patency.

There are few literatures that reported the synthesis of chitosan-peptide copolymers *via* point-to-point reaction. Chitosan, a deacetylated polysaccharide chitin, is usually hardly dissolved in neutral water and in most organic solvents, which makes liquid-phase reactions difficult to be carried out. Many studies performed the conjugating *via* solid-liquid reaction. At first, a chitosan membrane was made to react with peptide or protein solutions. This method evaded the difficulty of choosing appropriate solvent but brought new problems. First of all, only the top layer of chitosan molecules participated in the reaction, which will definitely lead to uneven contact between molecules and low reaction efficiency. Additionally, the complex procedure of the chitosan membrane preparation, reaction, and collection is time-consuming and adverse to preparing nanoparticles. In this study, only one step reaction was needed, which was more convenient for chitosan and peptide conjugation. TGase catalyzed an acyl transfer reaction between the γ-carboxamide groups of peptide-bound glutamine residues and the primary amino groups of chitosan (Lu et al., 2010; Fan et al., 2014; Silva et al., 2020). TGase is safe and efficient so it is widely used in the food and biomaterials industry (Chan et al., 2010; Perez-Puyana et al., 2019; Berg et al., 2021). The reaction condition of TGase is also suitable for chitosan since the pH and temperature are favorable for the dissolution of chitosan. The REDV peptides were grafted on chitosan by a Schiff’s base bond, which was known could be cleaved under local acidic environments. However, the CS-REDV copolymer was synthesized under pH 5.0 condition, and the normal blood pH was 7.34–7.45. It is hard to formulate an extremely low pH condition on the blood contacting surface. In this study, the FTIR test and fluorescence spectrophotometer test demonstrated the successful synthesis of the chitosan-REDV copolymer. This specific point-to-point covalent bonding is very strong and thus the copolymer is very stable in most of the reaction conditions if without enzyme catalyst (Lu et al., 2010), which is beneficial to the following synthesis of nanoparticles.

In the previous study, we found that nanoparticles with a positive charge and relatively uniform size were more efficient in immobilizing to the surface of scaffolds and localizing VEGF (Devalliere et al., 2018). Here we got the positive charge with a zeta potential of ∼13 mV and a PDI of .11. The zeta potential was lower than that we got in the previous study (Devalliere et al., 2018). A possible reason is that the REDV peptide brought in negative charges. The other reasonable explanation is that we had a higher heparin/chitosan mass ratio in this study, which meant a higher amount of heparin loading in CS-REDV-Hep NPs since no free heparin was detected in the nanoparticle solution. The optimal size of nanoparticles designed for drug delivery is approximately 50–150 nm, which confers a high surface area-to-volume ratio (Wang et al., 2015; Devalliere et al., 2018). In this study, the size of the CS-REDV-Hep NPs was about 61.3 ± 18.3 nm, and combined with the zeta potential and PDI, suggesting an ideal nanoparticle design for drug delivery. That was demonstrated in the heparin and VEGF release tests (Figures 3, 4).

Heparin is the most commonly used molecule for TEVG surface modification with the purpose of anti-thrombosis. In this study, heparin was utilized as a VEGF carrier as well, since heparin can bind VEGF *via* the specific VEGF binding site. Surface modification with heparin can significantly improve the blood compatibility of vascular grafts over a long period of time (Yao et al., 2014; Zhang et al., 2021). Heparin can promote the growth of HUVECs, while moderately inhibiting the proliferation of vascular smooth muscle cells (Wang et al., 2019). The heparin and CS-REDV copolymer formed nanoparticles with physical self-assembly *via* the opposite charges they have. This electrostatic connection can help to protect the biological activities of the molecules. Additionally, the whole system was composed of biomaterials without any cytotoxicity or immunogenicity, which may contribute to the good biocompatibility of the grafts. We detected a heparin loading rate of almost 100% in the nanoparticles. The 5/2 mass ratio of CS-REDV/Heparin seemed to be the utmost of heparin input because we observed aggregation immediately after a very small extra volume of heparin solution was put into the reaction system. Many researchers reported EDC/NHS cross-linking for heparin loading. This method provides a strong multi-point collection between the azyl of the heparin chain and the carboxyl of polymers. However, the fixed heparin molecules can hardly release from the graft (Bao et al., 2021). Instead, the self-assembly method was beneficial to keep heparin molecules’ activity. Heparin was released from the graft with the slow swelling of nanoparticles when in contact with water or blood and formed a concentration gradient around the lumen surface, which was critical for anti-thrombosis. In a recent study, Dongfang Wang reported self-assembly and deposition of layers of VEGF and heparin alternatively through electrostatic adsorption and cross-linked by genipin, and achieved a programmed release of VEGF and heparin *in vitro* triggered by matrix metallopeptidase 2 (Hamley, 2017). They demonstrated a sustained release of heparin and VEGF for 50 and 12 days, respectively. However, there were over 90% of the two molecules released from the grafts, which was much higher than that in this study. That may be attributed to the micro-structure of nanoparticles which enhanced the electrostatic adsorption between CS-REDV copolymer and heparin. The *in vitro* platelets adhesion assay demonstrated a low affinity for platelets adhesion and activity, which was the initial factor of thrombosis. The following *in vivo* implantation exhibited no thrombosis as well. These results confirmed a significant anti-thrombosis property of CS-REDV-Hep nanoparticles.

Chitosan alone has no specific cell adhesion activity. There are a massive number of azyl and carboxyl groups on the side chain of chitosan, and that makes chitosan an excellent platform for bioactive molecules, especially for peptide ligands. The REDV peptide is well acknowledged as the ligand of α4β1 integrin (Shan et al., 2018). Many *in vitro* investigations have been performed on REDV-immobilized surfaces and demonstrated good results (Jia et al., 2016; Mahara et al., 2020a; Zhou et al., 2021). REDV was demonstrated to be with a great affinity for ECs and EPCs (Mahara et al., 2015; Jia et al., 2016; Mahara et al., 2019; Mahara et al., 2020a; Zhou et al., 2021). Tetsuji Yamaoka et al. modified both acellular xenograft and synthetic materials with REDV peptide and tested them in small-animal and preclinical large animal transplantation models (Yamanaka et al., 2018; Mahara et al., 2019; Zhou et al., 2019; Mahara et al., 2020b). The REDV peptide on the luminal surface was found to not only capture REDV-specific cells in the circulation but also inhibit microthrombus formation *in vivo* (Mahara et al., 2015; Mahara et al., 2019). As a result, the REDV-conjugated grafts showed rapid endothelialization and stable short-term patency. In this study, we designed the peptide sequence QREDVY to conjugate with chitosan *via* the enzyme catalyze method, since the glutamine (Q) contains an amide residue that can react with the amino residues of chitosan. Furthermore, the tyrosine residues (Y) can emit autofluorescence under the effect of an appropriate excitation wave, which was convenient and accurate for quantification. We got a REDV/chitosan molecule ratio of 6.83%, which meant that every 100 chitosan repeat units can be grafted with approximately 7.0 REDV peptides. Considering the large amount of chitosan we used to form nanoparticles, we introduced abundant REDV peptide onto the luminal surface of the TEVG which resulted in better ECs adhesion *in vitro* (Figure 7G) and rapid endothelialization *in vivo*(Figure 10; Supplementary Figure S2).

Rapid and complete endothelialization is the key to achieving long-term patency no matter what kind of materials were used to fabricate small-diameter TEVG. Strategies to support the rapid growth of an ECs layer have been investigated for decades. One of the most commonly used molecules to promote the speed and level of endothelialization is VEGF. VEGF can bind specifically to the VEGF receptor (VEGFR) on the cytomembrane to promote ECs adhesion, proliferation, differentiation, migration, and vascularization by the VEGF/VEGFR-2 signal pathway (). In our previous study, VEGF was loaded on the bovine jugular vein graft and was proved to be beneficial for angiogenesis *via* a rat subcutaneous embedding model (Devalliere et al., 2018). However, when tested in a graft implantation model in rabbits, the ECs layer was still incomplete after 3 months. The result indicated that VEGF alone might not be adequate for rapid endothelialization. In this study, the CS-REDV-Hep Nps-VEGF system was designed to solve this problem. At the early stage of implantation, heparin was released from the graft and played an irreplaceable role in anti-platelet adhesion, aggregation, and anti-thrombosis. The REDV peptide captured circulating ECs/EPCs and promoted the initiation of endothelialization as early as the first day after implantation (Supplementary Figure S2). After that, the adhered cells were stimulated by VEGF through VEGF/VEGFR2 single pathway and proliferated to cover the luminal surface of TEVGs (Figures 10, 11). This method is of great value for TEVG investigation, and has the potential for transformation from “lab” to “bed.”

In the animal study, we found severe neo-intimal formation in the DP-BIMA group and mild neo-intimal formation in nanoparticle immobilized grafts, similar to Mahara described in their recent studies (Mahara et al., 2019; Zhou et al., 2019). Although there’s no occlusion happened in the long-term group of DP-Nps-VEGF immobilized grafts, neo-intimal formation-related stenosis was still be observed. Considering there’s no occlusion was found from 1 week to 3 months, we hypothesized that the regenerated ECs layer prevented the further narrowing of the grafts. However, if the endothelialization was delayed due to some specific conditions, the hyperplasia of the neo-intimal might continue and activate thrombogenesis to obstruct the whole graft lumen. Furthermore, the formation of neo-intimal may affect the bioactivity of surface modification. Further studies should be focused on this problem to get a more clear understanding of the mechanism of neo-intimal formation, and the strategies for inducing protein adsorption and neo-intimal formation should be investigated.

## Conclusion

In this study, a novel multi-functional nanoparticle-VEGF system that possesses sustained anti-thrombosis property, ECs/EPCs capture property, and ECs proliferation promoting property was successfully fabricated and immobilized on the luminal surface of decellularized BIMA. A patency rate of 100% and 83.3% for 1 and 3 months was achieved in the rabbit abdominal aorta interposition model. The *in vitro* and *in vivo* tests demonstrated good biocompatibility of the DP-Nps-VEGF immobilized BIMA and revealed good anti-thrombosis properties and rapid endothelialization which resulted in graft patency in a multi-functional system manner.

## Data Availability

The raw data supporting the conclusion of this article will be made available by the authors, without undue reservation.

## Abbreviations

BIMA, bovine internal mammary arteries; CS, chitosan; DP-BIMA, decellularized and photo-oxidized bovine internal mammary arteries; DP-Nps-BIMA, nanoparticle-immobilized DP-BIMA; DP-Nps-VEGF-BIMA, nanoparticle and VEGF immobilized DP-BIMA; EC, endothelial cell; EPC, endothelial progenitor cell; FTIR, fourier transform infrared spectroscopy; HUVECs, human umbilical vein endothelial cells; PDI, polymer dispersity index; QREDVY, Gln-Arg-Glu-Asp-Val-Tyr peptide; REDV, Arg-Glu-Asp-Val peptide; TGase, transglutaminase; TEVG, tissue-engineered vascular graft.
