# Supplementary material for: Multifunctional nanoparticle-VEGF modification for tissue-engineered vascular graft to promote sustained anti-thrombosis and rapid endothelialization
Source: Front Bioeng Biotechnol. 2023 Jan 17;11:1109058. doi: 10.3389/fbioe.2023.1109058 (PMC9887191; doi:10.3389/fbioe.2023.1109058)
Supplement: Supplementary file 1 [file Table1.docx]

| **Supplementary Table 1. The results of Hemolysis test of DP-Nps-VEGF-BIMA** | | | | | |
| --- | --- | --- | --- | --- | --- |
| **Class** | **Sample** | | | **X±S2** | **Hemolytic rate** |
|  | 1 | 2 | 3 |  |  |
| Dnc | 0.0004 | 0.0011 | 0.0006 | 0.0007±0.0003 | 0 |
| Dpc | 0.5046 | 0.5108 | 0.5095 | 0.5083±0.0033 | 1 |
| Dt | 0.0017 | 0.0005 | 0.0008 | 0.0010±0.0006 | 0.05% |

**Notes:** Dnc=Negative control, Dpc=Positive control, and Dt=DP-Nps-VEGF-BIMA samples. Dpc=0 indicated the test was effective. Dt<5% indicated good hemocompatibility of the materials.
