# Supplementary material for: Multifunctional nanoparticle-VEGF modification for tissue-engineered vascular graft to promote sustained anti-thrombosis and rapid endothelialization
Source: Front Bioeng Biotechnol. 2023 Jan 17;11:1109058. doi: 10.3389/fbioe.2023.1109058 (PMC9887191; doi:10.3389/fbioe.2023.1109058)
Supplement: Supplementary file 2 [file DataSheet1.docx]

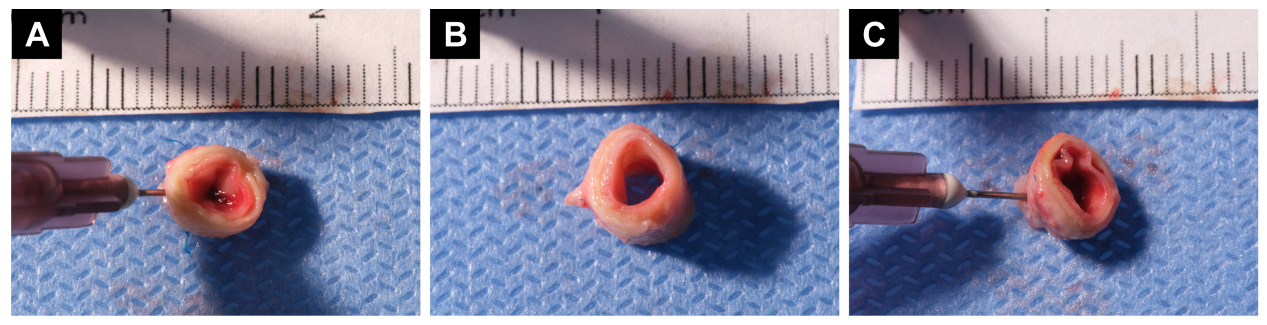


**Supplementary Figure 1.** A nanoparticle immobilized graft among the long-term samples was almost occluded at day 7. The neointimal of this graft was thicker, both at the proximal (A), middle (B), and the distal segment (C).


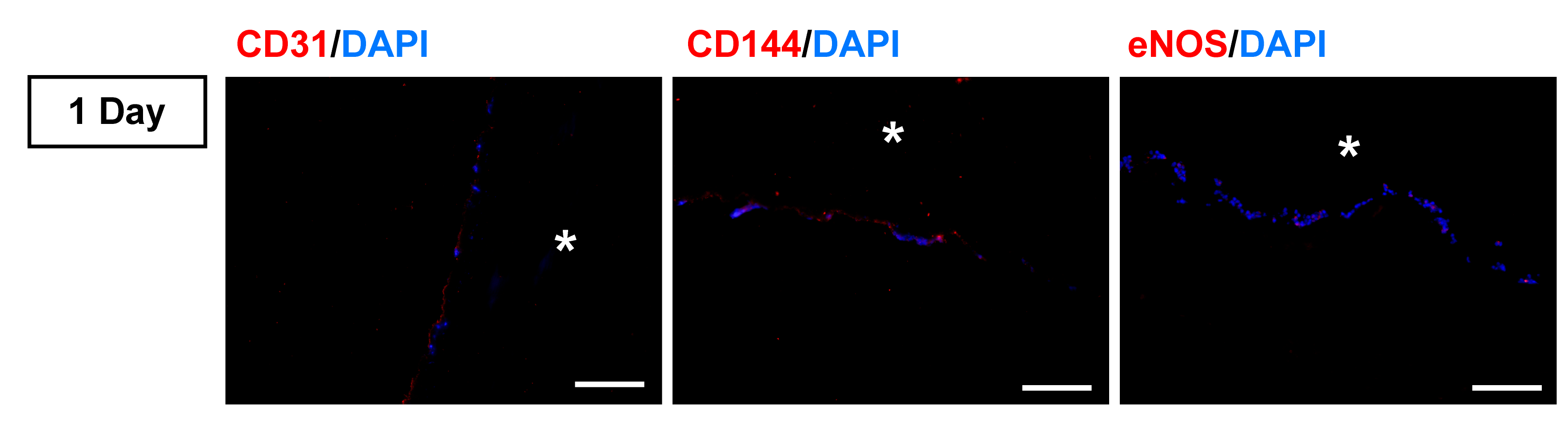


**Supplementary Figure 2.**The [immunofluorescent](D:/Program%20Files%20(x86)/Youdao/Dict/8.10.3.0/resultui/html/index.html#/javascript:;) [staining](D:/Program%20Files%20(x86)/Youdao/Dict/8.10.3.0/resultui/html/index.html#/javascript:;) results of CD31, CD144, and eNOS. The sample is a nanoparticle immobilized graft which was implanted for 1 day in the rabbit aorta replacement model. * indicates the lumen of the grafts. Scale bars indicate 100μm.


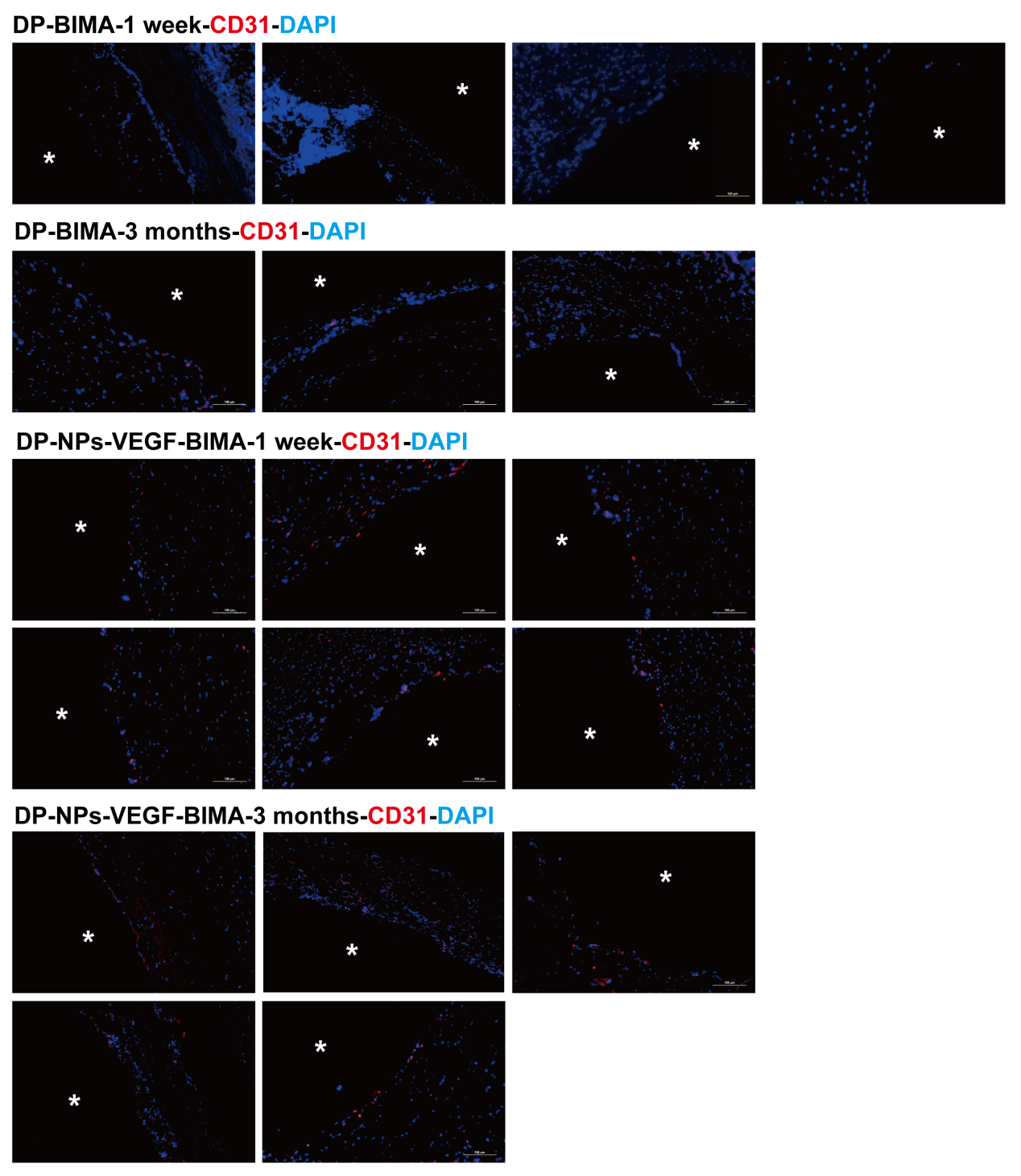


**Supplementary Figure 3.**The [immunofluorescent](D:/Program%20Files%20(x86)/Youdao/Dict/8.10.3.0/resultui/html/index.html#/javascript:;) [staining](D:/Program%20Files%20(x86)/Youdao/Dict/8.10.3.0/resultui/html/index.html#/javascript:;) results of CD31 staining for all the patent sample of each group. * indicates the lumen of the grafts. Scale bars indicate 100μm.


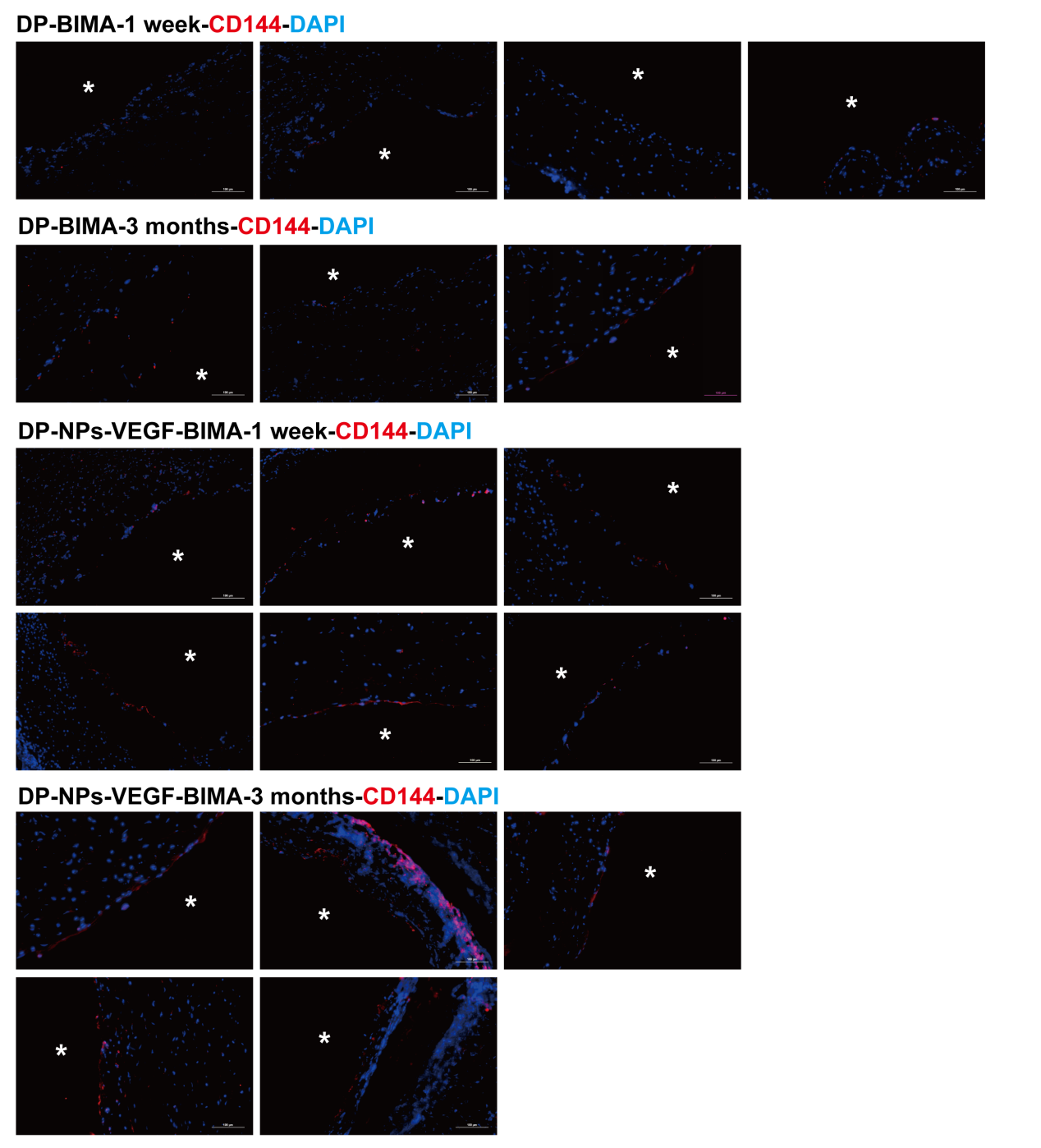


**Supplementary Figure 4.**The [immunofluorescent](D:/Program%20Files%20(x86)/Youdao/Dict/8.10.3.0/resultui/html/index.html#/javascript:;) [staining](D:/Program%20Files%20(x86)/Youdao/Dict/8.10.3.0/resultui/html/index.html#/javascript:;) results of CD144 staining for all the patent sample of each group. * indicates the lumen of the grafts. Scale bars indicate 100μm.


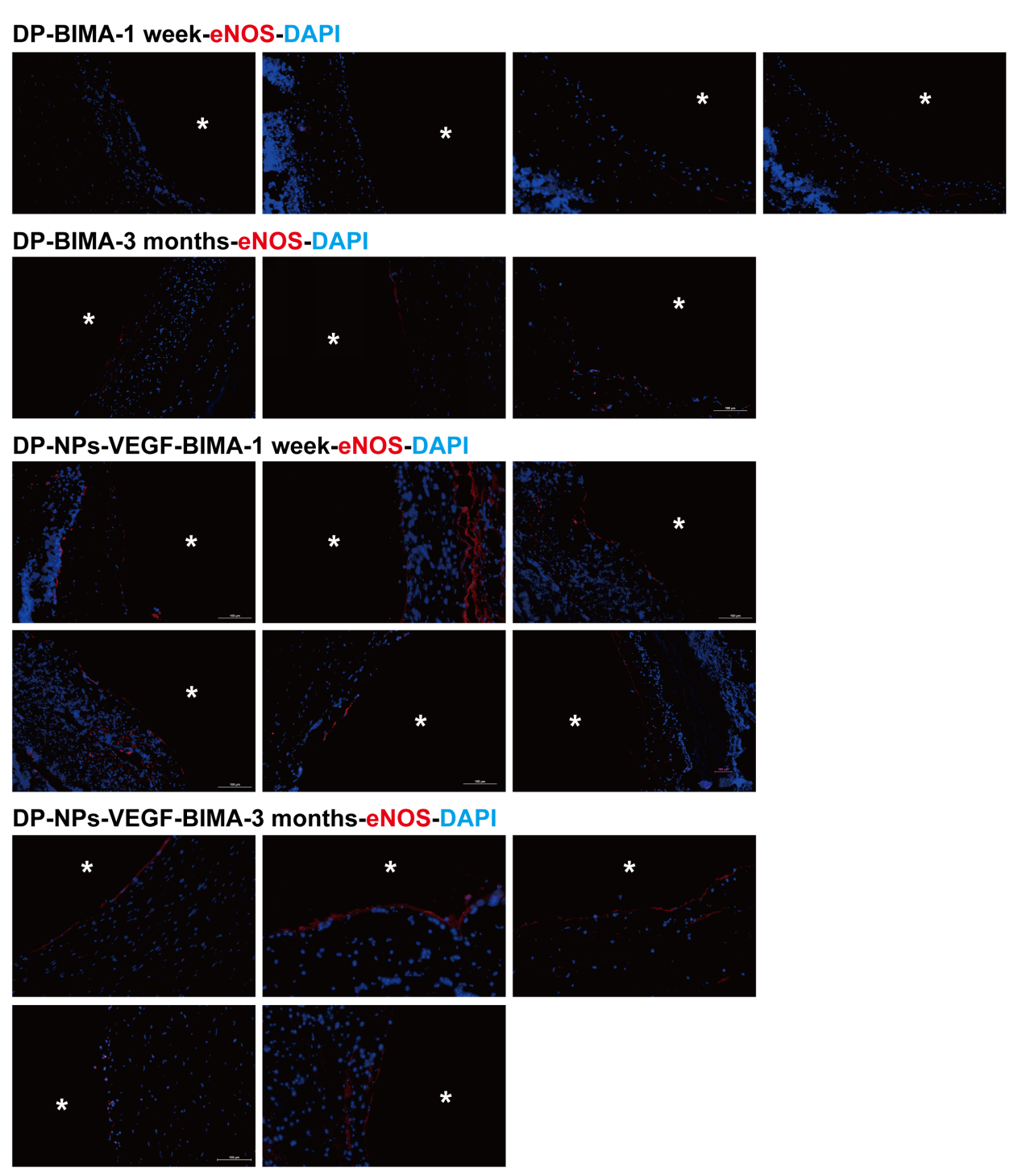


**Supplementary Figure 5.**The [immunofluorescent](D:/Program%20Files%20(x86)/Youdao/Dict/8.10.3.0/resultui/html/index.html#/javascript:;) [staining](D:/Program%20Files%20(x86)/Youdao/Dict/8.10.3.0/resultui/html/index.html#/javascript:;) results of eNOS staining for all the patent sample of each group. * indicates the lumen of the grafts. Scale bars indicate 100μm.
